# Further Studies on *Arcanobacterium phocisimile*: a Novel Species of Genus *Arcanobacterium*

**DOI:** 10.1155/2014/923592

**Published:** 2014-02-06

**Authors:** Osama Sammra, Anna Balbutskaya, Muaz Hijazin, Samy Nagib, Jörg Alber, Christoph Lämmler, Amir Abdulmawjood, Ellen Prenger-Berninghoff, Markus Timke, Markus Kostrzewa, Ursula Siebert

**Affiliations:** ^1^Institut für Pharmakologie und Toxikologie, Justus-Liebig-Universität Gießen, Schubertstraße 81, 35392 Gießen, Germany; ^2^Institut für Lebensmittelqualität und -sicherheit, Stiftung Tierärztliche Hochschule Hannover, Bischofsholer Damm 15, 30173 Hannover, Germany; ^3^Institut für Hygiene und Infektionskrankheiten der Tiere, Justus-Liebig-Universität, Frankfurterstraße 85-91, 35392 Gießen, Germany; ^4^Entwicklung Bioanalyse, Bruker Daltonik GmbH, Fahrenheitstraße 4, 28359 Bremen, Germany; ^5^Institut für Terrestrische und Aquatische Wildtierforschung, Stiftung Tierärztliche Hochschule Hannover, Bischofsholer Damm 15, 30173 Hannover, Germany

## Abstract

*Arcanobacterium phocisimile*, a newly described species with the type strain *A. phocisimile* 2698^T^ isolated from a vaginal swab of a harbour seal and four additional *A. phocisimile* strains also isolated from four harbour seals could reliably be identified by phenotypic properties, by matrix assisted laser desorption ionization time of flight mass spectrometry (MALDI-TOF MS), and by sequencing the genomic targets 16S rDNA and 16S-23S rDNA intergenic spacer region and the genes *rpoB* and *gap*. The *A. phocisimile* strains investigated in the present study were isolated together with several other bacterial species indicating that the pathogenic importance of *A. phocisimile* remains unclear. However, the detection of peptidic spectra by MALDI-TOF MS and the presented phenotypic and genotypic approach might help to identify *A. phocisimile* in future.

## 1. Introduction

Genus *Arcanobacterium* comprises the species *Arcanobacterium haemolyticum, Arcanobacterium hippocoleae, Arcanobacterium pluranimalium,* and *Arcanobacterium phocae* [1]. More recently *A. canis* and *A. phocisimile* were described as novel species of this genus [2, 3]. *Arcanobacterium pyogenes* together with *Arcanobacterium bernardiae, Arcanobacterium bonasi,* and *Arcanobacterium bialowiezense* was reclassified to the newly described species *Trueperella* [1]. The original description of *A. phocisimile* was based on physiological and biochemical characteristics, chemotaxonomic analysis, and 16S rDNA sequencing results of two strains isolated with several other bacterial species from a vaginal swab and an anal swab of two free living harbour seals of the German North Sea [3].

In the present study both initially described *A. phocisimile* strains and three additional strains obtained from three harbour seals were identified and further characterized phenotypically by MALDI-TOF MS analysis and genotypically by amplification and sequencing of various molecular targets.

## 2. Materials and Methods

### 2.1. Bacterial Strains

The *A. phocisimile* strains used in the present study included the previously described type strains *A. phocisimile*  2698^T^ (LMG 27073^T^; CCM 8430^T^) and *A. phocisimile* 4112 [3]. Additionally investigated *A. phocisimile* 3047 was isolated (post mortem) together with *Bacillus* spp., *Enterococcus* spp., *Erysipelothrix rhusiopathiae,* and  *α*-haemolytic streptococci in the year 2005 from the lung of a female harbour seal with bronchopneumonia and perforation of stomach. The harbour seal was found dead in Rantum, Sylt of the German North Sea. Also investigated *A. phocisimile* 4113 was recovered together with *Pasteurella* spp. and *α*-haemolytic streptococci from an anal swab and *A. phocisimile* 4125 together with *Pseudomonas* spp. and *α*-haemolytic streptococci also from an anal swab of two apparently healthy female harbour seals, respectively. *A. phocisimile* 4113 and *A. phocisimile* 4125 were isolated in 2007 during a monitoring program of free living harbour seals of the German North Sea.

### 2.2. Phenotypic and Genotypic Identification

All three newly investigated *A. phocisimile* strains were initially characterized phenotypically and by 16S rDNA sequencing [3, 4]. Both *A. phocisimile* strains previously mentioned in the species description [3] and the three *A. phocisimile* strains of the present study were further analysed by MALDI-TOF MS [5] and genotypically by amplification and sequencing of the previously described molecular target 16S-23S rDNA intergenic spacer region (ISR) and the genes *rpoB* and *gap* [4, 6, 7]. The primer sequences and the thermocycler programs are given in Table 1.

## 3. Results and Discussion

All three strains newly characterized in the present study could reliably be identified as *A. phocisimile* by phenotypic properties and by 16S rDNA sequencing. The phenotypic properties appeared to be almost identical to both previously characterized *A. phocisimile* strains (Table 2). However, a positive pyrazinamidase reaction of *A. phocisimile* seems to be the only reliable biochemical property for differentiation of *A. phocisimile* from pyrazinamidase negative *A. phocae*.

**Figure 1:**
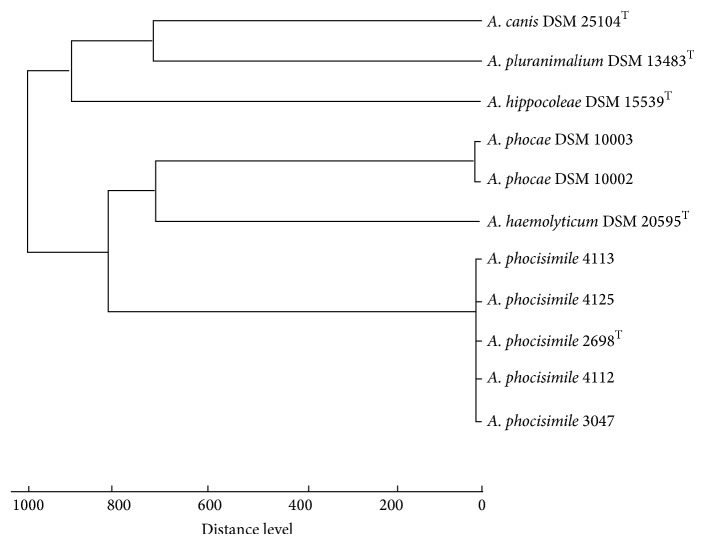
MALDI-TOF MS spectra from *A. phocisimile *4113, *A. phocisimile* 4125, type strain *A. phocisimile*  2698^T^, *A. phocisimile *4112, *A. phocisimile* 3047, and all other species of genus *Arcanobacterium*.

As shown by numerous authors MALDI-TOF MS is a powerful tool for species characterization of a broad spectrum of gram-positive and gram-negative bacteria [8–10]. This technique had previously been successfully used for rapid and reliable identification of bacteria of genera *Arcanobacterium* and *Trueperella* [5, 11]. The MALDI-TOF MS analysis of the present study revealed that by using the current Bruker data base, all five strains of this hitherto unknown species could not be identified to species level. However, using the MALDI Biotyper 3.1 software package the log (score) values of *A. phocisimile* 4112, *A. phocisimile* 3047, *A. phocisimile* 4113, and *A. phocisimile* 4125 matched against *A. phocisimile*  2698^T^ with log (score) values between 2.69 and 2.74 indicating that all five strains belong to this newly described species. Inclusion of *A. phocisimile* in the Bruker reference database will allow for the identification of this new species in future. A dendrogram analysis of the MALDI-TOF MS results is presented in Figure 1.

**Figure 2:**
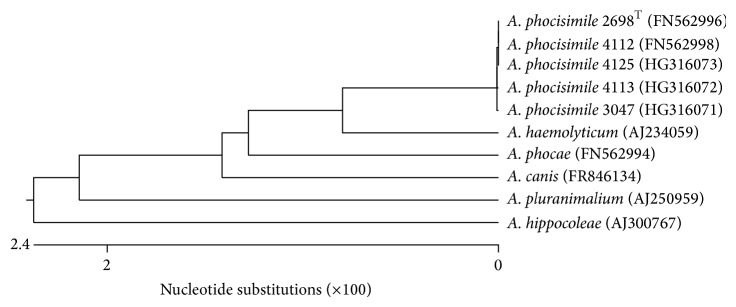
Dendrogram analysis of 16S rDNA sequences of the *A. phocisimile* strains of the present study and reference strains of genus *Arcanobacterium *obtained from NCBI GenBank.

The genotypic classification by 16S rDNA sequencing revealed that the three novel *A. phocisimile* strains of the present study yielded 100% identity to both *A. phocisimile* strains described previously [3], also including the type strain *A. phocisimile*  2698^T^ (Figure 2).

**Figure 3:**
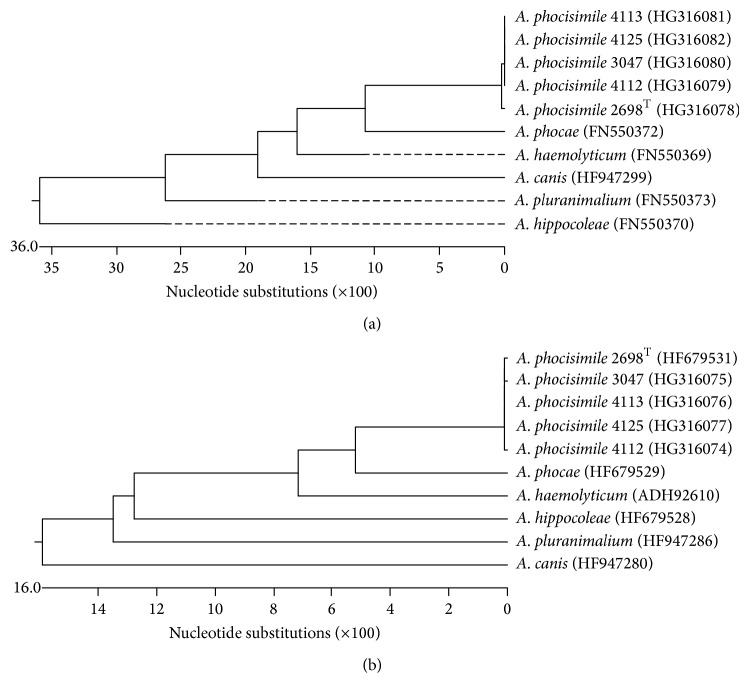
Dendrogram analysis of sequences of the genes *rpoB* (a) and *gap* (b) of the *A. phocisimile* strains of the present study and all other species of genus *Arcanobacterium* obtained from NCBI GenBank.

Comparable to previously described *A. canis* [11] all five *A. phocisimile* from the present study could additionally be classified by amplification and sequencing of ISR (FN563000, FN563002, HG316083, HG316084, and HG316085), gene *rpoB* (HG316078, HG316079, HG316080, HG316081, and HG316082), and gene *gap* (HF679531, HG316074, HG316075, HG316076, and HG316077) yielding for all three molecular targets an identity of ≥99.4%, ≥99.8%, and ≥99.8%, respectively, for all five strains among each other. A typical dendrogram using the sequencing results of the target genes *rpoB* and *gap* is shown in Figure 3.

The results of the present study revealed that phenotypic properties, the determination of peptidic spectra by MALDI-TOF MS, and the various genotypic targets allow for a reliable identification of *A. phocisimile* and a further differentiation of *A. phocisimile* from closely related *A. phocae* which could also be isolated from marine mammals [12]. However, all *A. phocisimile* strains of the present study were isolated together with various other bacteria, partly from obviously healthy animals, indicating that the pathogenic importance of this species for marine mammals remains unclear.

## 4. Interpretive Summary

*Arcanobacterium phocisimile* type strain and four additional *A. phocisimile* strains isolated from harbour seals were identified phenotypically, by matrix assisted laser desorption ionization time of flight mass spectrometry (MALDI-TOF MS), by sequencing 16S rDNA, and, as novel molecular targets, by sequencing 16S-23S rDNA intergenic spacer region and the genes *rpoB* and *gap* indicating that MALDI TOF MS and the molecular targets might help to identify this novel species.

## Figures and Tables

**Table 1:** Oligonucleotide primer sequences and PCR conditions of the target genes used in the present study.

Oligonucleotide primers	Sequence	Program∗	Expected size of PCR product (bp)	References
(1) 16S rDNA UNI-L (2) 16S rDNA UNI-R (amplification primer)	5′-AGAGTTTGATCATGGCTCAG-3′ 5′-GTGTGACGGGCGGTGTGTAC-3′	1	1,403	[4]
(3) 16S rDNA-533F (4) 16S rDNA-907R (sequencing primer)	5′-GTGCCAGCMGCCGCGGTAA-3′ 5′-CCGTCAATTCMTTTGAGTTT-3′	—	—	[4]
(5) Gap-F (6) Gap-R (*gap* primer)	5′-TCGAAGTTGTTGCAGTTAACGA-3′ 5′-CCATTCGTTGTCGTACCAAG-3′	2	830	[4]
(7) ISR-23S-F (8) ISR-23S-R (intergenic spacer region primer)	5′-CCTAGCCTGGTGGTTGGGTAG-3′ 5′-GTGCGGGTAACCAGAAATAACTCTG-3′	3	345	[6]
(9) C2700F (10) C3130R (*rpoB* primer)	5′-CGWATGAACATYGGBCAGGT-3′ 5′-TCCATYTCRCCRAARCGCTG-3′	4	406	[7]

^*^PCR program 1: x1 (95°C, 600 sec), x30 (95°C, 30 sec, 58°C, 60 sec, 72°C, 60 sec), and x1 (72°C, 420 sec). 2: x1 (94°C, 180 sec), x30 (94°C, 30 sec, 50°C, 40 sec, 72°C, 60 sec), and x1 (72°C, 300 sec). 3: x1 (95°C, 240 sec), x30 (95°C, 8 sec, 66°C, 10 sec, 72°C, 10 sec), x1 (72°C, 420 sec). 4: x1 (95°C, 600 sec), x35 (94°C, 30 sec, 50°C, 30 sec, 72°C, 120 sec), and x1 (72°C, 600 sec).

**Table 2:** Phenotypical properties of three *A. phocisimile* strains investigated in the present study and *A. phocisimile* 4112 and *A. phocisimile *2698^T^ described previously.

Phenotypic properties	3047	4113	4125	4112^**^	2698^T^^**^
Hemolysis on sheep blood agar	+	+	+	+	+
Hemolysis on rabbit blood agar	+	+	+	+	+
CAMP-like reaction with:∗					
*Staphylococcus aureus β*-hemolysin	−	−	−	−	−
*Streptococcus agalactiae *	+	+	+	+	+
*Rhodococcus equi *	+	+	+	+	+
*Arcanobacterium haemolyticum *	−	−	−	−	−
Reverse CAMP reaction	+	+	+	+	+
Nitrate reduction	−^1^	−^1^	−^1^	−^1^	−^1^
Pyrazinamidase	(+)^1^	(+)^1^	+^1^	+^1^	+^1^
Pyrrolidonyl arylamidase	−^1^	−^1^	(+)^1^	−^1^	−^1^
Alkaline phosphatase	−^1^	−^1^	−^1^	(+)^1^	(+)^1^
*β*-Glucuronidase (*β*-GUR)	−^1,3^	−^1,3^	−^1,3^	−^1,3^	−^1,3^
*β*-Galactosidase (*β*-GAL)	+^1,3^	+^1,3^	+^1,3^	+^1,3^	+^1,3^
*α*-Glucosidase (*α*-GLU)	+^1,2,3^	+^1,2,3^	+^1,2,3^	+^1,2,3^	+^1,2,3^
*β*-Glucosidase (*β*-GLU)	−^2^	−^2^	−^2^	−^2^	−^2^
N-Acetyl-*β*-glucosaminidase (*β*-NAG)	−^1^, +^3^	−^1^, +^3^	−^1^, +^3^	−^1^, +^3^	−^1^, +^3^
Esculin (*β*-glucosidase)	−^1^	−^1^	−^1^	−^1^	−^1^
Urease	−^1^	−^1^	+^1^	−^1^	−^1^
Gelatine	−^1^	−^1^	−^1^	−^1^	−^1^
Fermentation of:					
D-Glucose	+^1^	+^1^	+^1^	+^1^	+^1^
D-Ribose	−^1^	+^1^	+^1^	+^1^	+^1^
D-Xylose	−^1^	−^1^	−^1^	−^1^	−^1^
D-Mannitol	−^1^	−^1^	−^1^	−^1^	−^1^
D-Maltose	+^1^	+^1^	+^1^	+^1^	+^1^
D-Lactose	+^1^	+^1^	+^1^	+^1^	+^1^
D-Saccharose	+^1^	+^1^	+^1^	+^1^	+^1^
Glycogen	+^1^	+^1^	+^1^	+^1^	+^1^
*α*-Mannosidase	+^2^	+^2^	+^2^	+^2^	+^2^
Catalase	+	+	+	+	+
Serolysis on Loeffler agar	−	−	−	−	−
Caseinase	−	−	−	−	−
Starch hydrolysis	+	+	+	+	+

The reactions are shown as follows: ^*^synergistic CAMP-like reaction with indicator strains; ^**^results mostly obtained from Hijazin et al., 2013 [3]; +: positive reaction; (+): weak positive reaction; −: negative reaction; 1: Api Coryne test system (Biomerieux, Nürtingen, Germany); 2: tablets containing substrates (Rosco Diagnostica A/S, Taastrup, Denmark); 3: 4-methylumbelliferyl conjugated substrates (Sigma, Steinheim, Germany).
